# Value-based sleep and breathing: health economic aspects of obstructive sleep apnea

**DOI:** 10.12703/r/10-40

**Published:** 2021-04-19

**Authors:** Emerson M Wickwire

**Affiliations:** 1 Sleep Disorders Center, Division of Pulmonary and Critical Care Medicine, Department of Medicine, University of Maryland School of Medicine, Baltimore, MD, USA; Department of Psychiatry, University of Maryland School of Medicine, Baltimore, MD, USA

**Keywords:** Sleep, sleep apnea, treatment, economics, costs, cost-effectiveness

## Abstract

Obstructive sleep apnea (OSA) is a common and costly medical condition. Untreated OSA is associated with numerous and well-documented adverse health consequences including depression, diabetes, cardiovascular disease, and premature death. In addition to these health consequences, untreated OSA is also associated with substantial costs borne by patients, payers, the health system, and society at large. Perhaps more importantly, evidence suggests that OSA treatment is associated with positive economic benefit. The purpose of this brief review is to introduce economic aspects of OSA, including the potential economic benefit of OSA treatment.

## Introduction

Obstructive sleep apnea (OSA) is a common and costly medical condition. The health consequences of OSA include increased risk for cardiovascular disease^1–3^, stroke^4^, metabolic syndrome^5,6^, reduced quality of life^7^, and premature death^8,9^. Approximately 14% of men and 5% of women between the ages of 30 and 70 years in the United States suffer moderate to severe OSA^10^, with 936 million individuals estimated to suffer OSA worldwide^11^. In part because of rising rates of obesity, the prevalence of the disorder is increasing^12,13^.

Beyond well-documented adverse health outcomes, OSA incurs dramatic economic costs borne by patients, payers, employers, and society^14^. Yet, despite widespread acknowledgment that OSA is associated with substantial economic burden, relatively little research has evaluated economic aspects of OSA treatments^15^. In the modern healthcare climate of rising costs on the one hand and limited resources on the other, such knowledge is essential for payers and policy-makers who seek to make evidence-based decisions regarding allocation of scarce healthcare resources. To this end, the purpose of the present paper is to introduce key health economic concepts pertaining to OSA and to very briefly summarize the evidence to date regarding health economic aspects of OSA.

## Very brief introduction into health economic aspects of obstructive sleep apnea

### Costs of obstructive sleep apnea

Several years ago, the American Academy of Sleep Medicine commissioned a white paper that estimated total societal-level costs of OSA to exceed $150 billion per year in the United States alone^16^. Broadly speaking, these OSA costs can be divided into three categories: direct costs, indirect costs, and health-related quality of life (HrQOL). Direct costs of OSA include costs of OSA diagnosis and management, including provider consultation, sleep apnea testing, device and supply costs (e.g. continuous positive airway pressure [CPAP] or oral appliance [OA] therapy), prescription medications, and so on. Indirect costs include costs unrelated to direct OSA care that are nonetheless attributable to OSA, such as increased healthcare utilization (HCU) for non-OSA conditions, diminished workplace productivity (e.g. absenteeism or presenteeism [being physically present but less productive than expected]), and increased risk of accidents and errors including motor vehicle collisions (MVCs). In the AASM white paper, the greatest costs associated with OSA were lost workplace productivity ($86.9 billion), increased HCU ($30 billion), MVCs ($26.2 billion), and workplace accidents and injuries ($6.5 billion)^16^. Although total societal-level costs are often cited to demonstrate the costs associated with OSA and other sleep disorders, it is important to realize that each of these costs is primarily borne by different stakeholders^17^.

### Health-related quality of life

In addition to direct and indirect costs that can be clearly monetized, HrQOL is a key economic outcome^18,19^. HrQOL includes both general as well as disease-specific outcomes, which can be measured using various tools. Common general HrQOL measures include the PROMIS, developed as part of the National Institutes of Health Roadmap^20^, Short-Form 36 (SF-36)^21^, EuroQol-5D^22^, and others; the Functional Outcomes of Sleep Questionnaire (FOSQ)^23^ is an example of an OSA-specific measure of HrQOL. The most common economic metric to quantify HrQOL is the quality adjusted life year (QALY). QALYs represent the product of HrQOL and time (i.e. [quality on 0–1 utility scale] * [time in years]) and thus provide researchers and decision-makers with a standardized metric to compare return-on-investment across diverse disease states, such as in cost-effectiveness analyses (CEAs)^15^. In CEAs, cost-effectiveness is determined based on the ratio of quality to cost, which is known as the incremental cost-effectiveness ratio (ICER). As a measure of value, ICERs reflect the cost per QALY. A given health system or payer determines a willingness to pay (WTP) for a QALY. For example, in the US, a cost of $50,000 per QALY is a generally accepted threshold for cost-effectiveness. Over the past three-plus decades, a preponderance of evidence demonstrates that untreated OSA worsens HrQOL and OSA treatments improve HrQOL and are generally cost-effective^14^.

### Economic perspective: value is in the eye of the beholder

The sleep medicine ecosystem includes diverse stakeholders such as patients, payers, providers, health systems, and equipment manufacturers, just to name a few. Each of these stakeholders incurs different costs (and potentially cost savings or revenues) so perceives the value of sleep differently. For example, patients desire improved quality of life, such as increased energy for work or leisure activities, as well as ease of care experience. Payers want to reduce costs of care delivery, which is directly related to profits. Providers want valid, reliable, and easy-to-administer testing and treatment options, and so on. Table 1 presents common economic perspectives in sleep medicine.

**Table 1.** Economic perspectives in sleep medicine.

Perspective	Value-based outcome
Patient	Quality of life, ease of treatment experience
Payer	Cost savings for increased profitability
Employer	Workplace productivity, accident risk
Health system	Revenue (margin), population health
Society	Aggregated costs and health economic outcomes

## Diagnosing and treating obstructive sleep apnea: economic considerations

From an economic perspective, the diagnosis of OSA involves two broad components: provider time and sleep apnea testing. As a result, the most expensive costs would include a face-to-face physician encounter, in regulated hospital space, and in-lab diagnostic polysomnography with an additional in-lab CPAP titration. At the other extreme, diagnostic costs could include consultation with a mid-level provider, via telehealth, and home sleep apnea testing (HSAT) without a separate titration study. Like OSA diagnosis, OSA treatment costs include two components: initial device and equipment (e.g. CPAP) and ongoing supply costs. Furthermore, it is important to realize the competing interests in these OSA pathways: cost savings (a positive) from the payer’s perspective results in reduced revenue (a negative) from the health system’s perspective or decreased reimbursement (a negative) from the provider’s perspective. As one example, consider findings from an economic analysis of a multi-site clinical trial designed to compare in-lab versus at-home approaches to OSA diagnosis and treatment^24^. Kim and colleagues found that payer costs were reduced by $264 over three months when patients were treated within the at-home arm (as opposed to the in-lab arm). By contrast, provider operating margin was reduced from $142 to –$161 under these same conditions. Finally, time horizon is an important consideration: most OSA treatment costs are front-loaded, but OSA cost reductions might not be realized immediately.

As healthcare transitions from volume-based care to value-based care, alternative payment models (APMs) have been employed in numerous disease states. To date, APMs have not yet impacted sleep medicine in the US in a meaningful way. Nonetheless, bundled payments and shared risk are likely to impact the field of sleep medicine sooner rather than later. Within a value-based framework, both maximizing quality and minimizing cost are essential. Such matters, including cost-shifting between stakeholders, have been discussed in detail elsewhere^17^.

## Economic impact of obstructive sleep apnea treatments

Relative to thousands of studies that evaluated pathophysiologic, clinical, and epidemiologic aspects of OSA, few studies have considered the economic impact of OSA treatments. Two prior reviews primarily considered costs of untreated OSA^25^ or included only a very limited number (n = 5) of studies that examined the impact of OSA treatment on HCU^26^. More recently, our group published a systematic review of the impact of OSA treatments on monetized economic outcomes. Results demonstrated that 15 of 17 comparisons reported a positive economic benefit from OSA treatment (results are included in Table 2)^14^. Since that time, two additional studies have found a beneficial effect on PAP. Kirsch and colleagues^27^ analyzed data from a large health system in the Southeastern US. These investigators found a linear dose-response relationship between objective CPAP adherence data and acute healthcare costs, including reductions in inpatient stays and acute care visits. A particular strength of this study was the use of objective CPAP adherence data, which enables the evaluation of dose-response effects. At the same time, although this study controlled for baseline differences between adherence groups, it was limited in its approach to control for the “healthy user effect” over time (i.e. that CPAP adherence is associated with positive health behaviors)^40,41^, which might have contributed to the positive findings. Similarly, Chhatre and colleagues analyzed data from a large national Medicare administrative claims database^32^. When the authors used CPAP machine charges as a proxy for objective CPAP use, CPAP was associated with reduced total costs. Interestingly, de Vries and colleagues^33^ found OA therapy more cost-effective than CPAP in terms of QALYs, but not in terms of reduction in apnea-hypopnea index, over 12 months. Results of these and other studies examining the economic impact of OSA treatments, reflecting a non-systematic update since our prior systematic review 14, are summarized in Table 2.

**Table 2.** Summary of empirical studies examining the impact of OSA treatments on monetized economic outcomes.

Ref	Sample	Design	OSA treatment	Economic outcome	Key findings
28	N = 34, M age = 48y, 100% men in Canada	Retrospective cohort study	CPAP	Outpatient visits, physician costs	Vs. 1y prediagnosis, CPAP reduced outpatient visits (1.03 visits) and physician costs by $14.23 over 5y
29	N = 344, M age = 49y, 100% men in Canada	Prospective cohort study	CPAP or BPAP	Physician costs, hospitalizations	Vs. 2y prediagnosis and among adherers, CPAP reduced physician costs and hospitalizations over 2y
30	N = 414, M age = 49y in Canada	Retrospective cohort study	CPAP or BPAP	Outpatient visits, physician costs	Vs. 1y prediagnosis, CPAP reduced outpatient visits and physician costs by $37.26 over 2y
31	N = 15,424, M age = 48y, 70% men in USA	Retrospective cohort study	CPAP	Total costs, all-cause and OSA-related hospitalizations	Vs. 1y prediagnosis, CPAP reduced total costs ($792 vs. $883) and rates of all-cause (19% vs. 24.2%) and OSA-related (8% vs. 11.3%) hospitalizations over 2y
32	N = 22,361 w/OSA, M age = 67.2y, 53% men in US	Retrospective cohort study	CPAP	Total costs	Vs. 1y prediagnosis, CPAP adherence reduced total costs
33	N = 86, M age = 50.7y, 82.3% men in US	Multicenter RCT	CPAP, OA	QALY, total costs	CPAP was more clinically effective, but based on cost per QALY, OA was more cost-effective at 12 months (€33.701 [−€191.106 to €562.271] per QALY gained)
34	N = 248, M age = 44y, 99% men commercial drivers in USA	Retrospective cohort study	CPAP or BPAP	Total costs	Vs. 1y prediagnosis, CPAP reduced HCU costs over 2y (y1: $3,062; y2: $3,465)
35	N = 30,719, M age = 67.1y, 43% men in USA	Retrospective cohort study	CPAP	Costs	Vs. those not tested, clinically diagnosed, and not treated ($12,080/quarter [$10,759 in 2010]), costs were lowest for those tested, diagnosed, and treated ($6,465/quarter [$5,758 in 2010])
36	N = 19,438, 78% men in Denmark	Retrospective cohort study	CPAP, UPPP	Total costs	Vs. 2y prediagnosis, neither CPAP nor UPPP reduced HCU costs over 2y
37	N = 1,098, M age = 55.7y, 63% men in US	Retrospective cohort study	CPAP	Acute care HCU and costs	CPAP adherence reduced inpatient (RR = 0.92, 95% CI: 0.86–0.98) and overall acute care visits (RR = 0.96, 95% CI: 0.92–0.99). Among CPAP adherers, fewer ED visits and inpatient stays were observed.
37	N = 278, M age = 71y, 79% men in UK	Multi-center RCT	APAP	CEA HrQOL: EQ-5D and SF-6D	Vs. untreated OSA, APAP was associated with 0.018 QALYs gained per SF-6D, but no gains per EQ-5D. APAP reduced costs (–$61 [–£35 in 2014 GBP]) over 1y and was marginally cost-effective.
38	N = 82, M age = 55y, 82% men in Sweden	Retrospective cohort study	CPAP	Hospital costs	Vs. 2y prediagnosis, CPAP reduced CVPD-related hospitalization costs ($80,680 vs. $11,134) over 2y
39	N = 740 children <18y, M age = 5.6y, 37% boys in Israel	Prospective, longitudinal case- control study	T&A	Total costs	Vs. 1y prediagnosis, CPAP reduced total costs (32.5%; *P* <0.0004) over 1y

APAP, automatic positive airway pressure; BPAP, bilevel positive airway pressure; CEA, cost-effectiveness analysis; CPAP, continuous positive airway pressure; CVPD, cardiovascular and pulmonary disease; ED, emergency department; EQ-5D, European Quality of Life 5 Dimension; HCU, healthcare utilization; HrQOL, health-related quality of life; OA, oral appliance; OSA, obstructive sleep apnea; QALY, quality adjusted life year; RCT, randomized controlled trial; RR, rate ratio; SF-6D, Short Form questionnaire-6 Dimensions; T&A, adenotonsillectomy; UPPP, uvulopalatopharyngoplasty

## Socioeconomic aspects of obstructive sleep apnea

Racial and socioeconomic factors have been identified as an important determinant of OSA and OSA outcomes. For example, relative to whites, blacks suffer higher rates of OSA but are less likely to be diagnosed^42^. Evidence suggests that such racial sleep disparities might underlie racial disparities in chronic disease outcomes, such as cardiovascular disease and diabetes^43^. Relative to whites, blacks are also less likely to adhere to CPAP therapy^44^, and low socioeconomic status (SES) and neighborhood of residence have also been associated with poorer CPAP adherence^45,46^. These results further support a link between OSA disparities and health disparities. In recognition of the importance of sleep disparities in overall health disparities, the National Institutes of Health recently hosted a workshop and identified research needs; a summary report has been published elsewhere and is recommended for review^47^.

## Future research directions

Our most important recommendation is to include economic outcomes in OSA clinical trials and sleep research more broadly. Public–private partnerships are likely to be especially fruitful in this regard. Second, in light of the high rates of comorbidity of OSA with numerous medical and psychiatric conditions (e.g. cardiovascular disease, depression, and neurodegenerative disorders, including Alzheimer’s disease), it will be important to evaluate economic aspects of OSA within various disease subpopulations. Disentangling the effects of OSA relative to the often better studied comorbid conditions, such as cardiovascular disease, is methodologically challenging yet vital^48^. Third, it is essential to adopt the employer’s perspective when assessing the economic impact of OSA. It is notable that the majority of OSA-related costs are borne by employers, yet very few studies have considered economic aspects of OSA from the employer’s perspective. Fourth, given the positive economic impact of CPAP adherence, much greater insight is needed regarding the costs and effectiveness of interventions to improve CPAP adherence^14,48,49^. Finally, given the very rapid technological advancements in OSA diagnosis and treatment, it will be important to evaluate the economic impact of telehealth and remote monitoring for OSA. This recommendation is especially important given the impact of the COVID-19 pandemic in terms of clinical care and rapid adoption of telehealth approaches. Additional actionable recommendations to advance understanding regarding the health economic aspects of OSA are presented in Table 3.

**Table 3.** Recommendations for future research in OSA.

Domain	Recommendation
Include health economic outcomes	Measure direct and indirect costs of OSA in OSA trials
Measure cost-effectiveness	Measure both general and disease-specific HrQOL in OSA trials
Study specific populations	Perform health economic analyses among demographic groups, including women, older adults, and children; among different racial groups; and among patients with varying OSA severity
Investigate comorbid OSA	Evaluate economic impact of OSA and OSA treatments in key comorbid subpopulations such as patients with heart failure, type 2 diabetes mellitus, and depression
Increase adherence	Study economic aspects of interventions to increase treatment adherence, including cognitive– behavioral treatment for CPAP, telehealth and remote monitoring, and automated approaches
Adopt employer perspective	Evaluate cost–benefit of OSA treatments from the employer’s perspective, including impact on workplace productivity as well as accident and injury risk
Consider global impact	Evaluate cost-effectiveness of treating OSA in various healthcare delivery systems globally
Compare economic effectiveness	Compare economic effectiveness of OSA treatments to enable evidence-based decision-making regarding allocation of limited healthcare resources

CPAP, continuous positive airway pressure; HrQOL, health-related quality of life; OSA, obstructive sleep apnea

## Conclusions

In summary, OSA incurs significant economic costs borne by a wide range of stakeholders including patients, payers, health systems, industry partners, and others. Although important questions remain unanswered, a small but considerable body of evidence suggests that the diagnosis and treatment of OSA is associated with positive economic benefit. Our most important recommendations are for researchers to include economic endpoints in all OSA clinical trials and to adopt the economic perspectives of multiple stakeholders in the sleep medicine ecosystem. In the modern healthcare climate of rising costs on one hand and limited resources on the other, economic aspects of care will become increasingly important determinants of health policy and resource allocation.
